# Use of Evidence-Informed Deliberative Processes by Health Technology Assessment Agencies Around the Globe

**DOI:** 10.15171/ijhpm.2019.72

**Published:** 2019-09-15

**Authors:** Wija Oortwijn, Maarten Jansen, Rob Baltussen

**Affiliations:** Department for Health Evidence, Radboud Institute for Health Sciences, Radboud University Medical Centre, Nijmegen, The Netherlands.

**Keywords:** Health Technology Assessment, Evidence-Informed Deliberative Processes, Legitimate, Decision-Making, Guidance

## Abstract

**Background:** Evidence-informed deliberative processes (EDPs) were recently introduced to guide health technology assessment (HTA) agencies to improve their processes towards more legitimate decision-making. The EDP framework provides guidance that covers the HTA process, ie, contextual factors, installation of an appraisal committee, selecting health technologies and criteria, assessment, appraisal, and communication and appeal. The aims of this study were to identify the level of use of EDPs by HTA agencies, identify their needs for guidance, and to learn about best practices.

**Methods:** A questionnaire for an online survey was developed based on the EDP framework, consisting of elements that reflect each part of the framework. The survey was sent to members of the International Network of Agencies for Health Technology Assessment (INAHTA). Two weeks following the invitation, a reminder was sent. The data collection took place between September-December 2018.

**Results:** Contact persons from 27 member agencies filled out the survey (response rate: 54%), of which 25 completed all questions. We found that contextual factors to support HTA development and the critical elements regarding conducting and reporting on HTA are overall in place. Respondents indicated that guidance was needed for specific elements related to selecting technologies and criteria, appraisal, and communication and appeal. With regard to best practices, the Canadian Agency for Drugs and Technologies and the National Institute for Health and Care Excellence (NICE, UK) were most often mentioned.

**Conclusion:** This is the first survey among HTA agencies regarding the use of EDPs and provides useful information for further developing a practical guide for HTA agencies around the globe. The results could support HTA agencies in improving their processes towards more legitimate decision-making, as they could serve as a baseline measurement for future monitoring and evaluation.

## Background

Health technology assessment (HTA) is used to inform decision-making, such as coverage-decision making, and is described as a process that includes governance and structure, scoping, assessment, appraisal and implementation and monitoring.^1^ There is broad recognition that current HTA processes are ill fitted to take into account the wide range and diversity of stakeholder values and lead to insufficient sets of information. Ethical issues in particular are left unaddressed, thereby compromising the legitimacy of eventual decisions. The call for a more integrative perspective on HTA aligns with evidence-informed deliberative processes (EDPs) that were recently introduced to support HTA agencies in organizing legitimate processes.^2,3^ Although the use of EDPs is relatively new, deliberative methods have been developed and used to some extent in the field of HTA since the 2000s.^4-9^

EDPs draw on this earlier work and provide a structured process in which stakeholders participate throughout the HTA processto identify criteria for the selection of health technologies and assessment, to interpret forthcoming evidence, and to deliberate on recommendations and decisions.^10,11^ EDPs are based on rational decision-making through evidence-informed evaluation of identified relevant values (reflected as criteria used in multi-criteria decision analyses [MCDAs]) as well as fair decision-making (as reflected in the accountability for reasonableness approach – A4R). The underlying premises of the EDP framework are: (1) that involvement of relevant stakeholders to identify, reflect, and learn about the meaning and importance of relevant values and questions, and (2) an evidence-informed evaluation of the identified values (criteria), can contribute to the legitimacy of recommendations and/or decisions by improving the quality, consistency and transparency of the HTA process.

Some HTA agencies already have several of these components in place (eg, the Canadian Agency for Drugs and Technologies in Health [CADTH], Scottish Medicines Consortium, National Institute for Health and Care Excellence [NICE] in the United Kingdom, and the National Committee for Health Technology Incorporation [CONITEC] in Brazil).^11^ Several approaches undertaken by these agencies rely on deliberative processes, for example regarding identifying topics for HTA and how to appraise health technologies. These agencies may serve as inspiration for others, especially those who have recently formally set-up their HTA practice (eg, the Ministry of Public Health in Uruguay, and the Centre of Standardization of the Republican Centre for Health Development in Kazakhstan), but they can also improve regarding certain components.^3^

In order to support HTA agencies in the use of EDPs, we recently developed a first version of a guide.^12^ In this practical guide, we describe the EDP framework that consists of 5 steps and contextual factors for HTA development (Figure).

**Figure:**
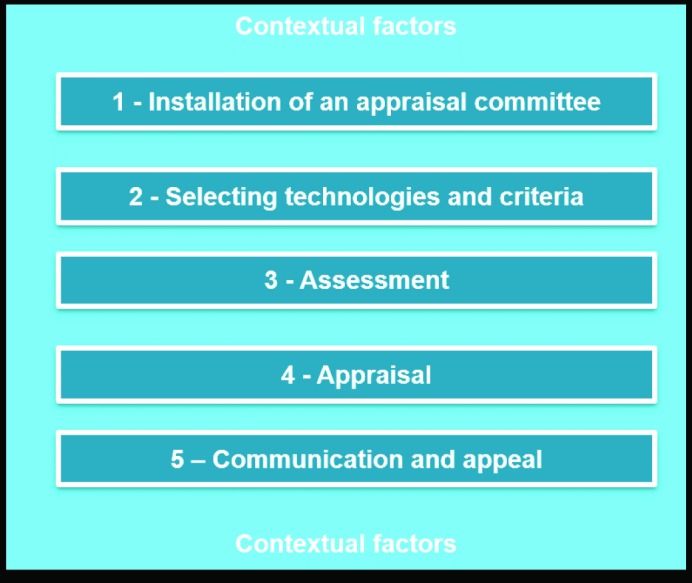
The Steps of EDPs. Abbreviation: EDPs, evidence-informed deliberative processes.

We defined the core elements of each step on the basis of our previous work in this field,^3,10^ and existing checklists such as the checklist for HTA reports from the International Network of Agencies for Health Technology Assessment (INAHTA).^13^ The way in which these steps can be applied in a specific country or region depends on the context, and therefore is not meant as a blueprint.

To identify the level of use of EDPs by HTA agencies, and to further develop the guide to support HTA agencies, we felt it was important to collect views and experiences from HTA agencies around the globe. We therefore conducted a survey among INAHTA member agencies to identify the current level of use of EDPs, identify the need for guidance regarding elements of the framework, and to learn about best practices. In this article, we present the main findings from the survey.

## Methods

A questionnaire for a semi-structured online survey was developed based on the EDP framework, consisting of elements that reflect each step of the framework and the contextual factors for HTA development. We also asked respondents to list any element that they felt was missing and for best practices regarding each part of the framework. The questionnaire is provided as Supplementary file 1.

We contacted the INAHTA secretariat for supporting us in contacting their members. INAHTA is a network of 50 HTA agencies that support health system decision-making in 31 countries around the globe (2018 figures). The INAHTA secretariat provided consent and agreed to send an invitation to participate in the survey to their members through email. Two weeks following the initial invitation, a reminder was sent by the research team, targeting the contact persons as listed on the INAHTA website, which is publicly available. A second reminder was sent 2 weeks after the first reminder. The data collection took place, using the online tool CheckMarket, between September-December 2018.

In the introduction to the survey, we made clear that all answers were treated confidential, ie, no attribution would be made to specific persons. Two survey respondents explicitly wished not to disclose their affiliation. Therefore, we provide the results anonymously, presenting the main results and highlighting specific areas that were felt to be in need of guidance. We used basic descriptive statistics (frequencies, presented as percentage), derived from the CheckMarket tool, to summarize the findings.

## Results

### Response Rate

Twenty-five completed survey forms were received from 15 European HTA agencies, 4 HTA agencies were located in the Asia-Pacific region (15%), 3 HTA agencies in Latin-America (11%), 2 came from North-America (7%) and 1 was situated in South-Africa (4%). We received incomplete survey responses from 5 other HTA agencies, of which we included the survey responses from 2 European HTA agencies that provided meaningful responses to some of the questions (63%). As the number of member agencies was 50 in 2018, the overall response rate was 54%. This is comparable with other studies that surveyed INAHTA members.^14,15^ In addition, the distribution of HTA agencies providing responses, per region can be considered a representative reflection of the INAHTA membership. From the 50 member agencies, 64% is located in Europe, 14% in the Asia-Pacific region, 10% in Latin-America as well as in North-America, and 2% in South-Africa; http://www.inahta.org.

### Contextual Factors

All agencies were asked about the presence of factors that are supportive of HTA development and whether these were in need of guidance in their specific context, ie, factors reflecting the linkage between HTA practice and policy; the level of institutionalization of HTA and the ability to networking and capacity building. Overall, the respondents mentioned that the contextual factors were present in their contexts, and up to 30% of the respondents felt that guidance was needed for specific factors (Table 1). There was no clear distinction between member agencies with a longer (well-established agencies; 93%) or shorter tradition (agencies that are new, in the process of being established, and/or are not yet producing HTA reports; and a member of INAHTA for less than 2 years) of HTA development (7%).

**Table 1:** Views Regarding the Presence of Contextual Factors and the Need for Guidance (n = 27)

**Contextual Factor**		**Present**	**Present to Some Extent**	**Not Present**	**Guidance Needed**
Linkage between HTA and practice/policy	A (formal) mechanism or process to link HTA to policy-making (eg, legislation)	52%	48%	0%	30%
	Allocation of public funding to HTA on an annual basis	74%	15%	11%	26%
	A policy statement on the willingness to use HTA in policy and/or practice	74%	22%	4%	26%
Institutional environment	An independent organizational structure and/or institutional set-up for HTA (HTA organization or HTA focal point)	78%	22%	0%	22%
	HTA process guidelines (is a systematic process in place eg, assessment and appraisal)	67%	30%	4%	26%
	HTA method guidelines (eg, for conducting economic analysis or clinical assessment)	67%	33%	0%	22%
Networking and capacity	An (inter)national networking strategy for collaboration between HTA organization(s) and relevant stakeholders	52%	33%	15%	30%
	Sufficient capacity to carry out HTA	52%	41%	7%	30%
	Ability to review international literature (ie, access to databases), including expertise in searching the internet	93%	7%	0%	4%
	(Domestic) HTA training opportunities (short courses, workshops, master programs and PhD training)	59%	33%	8%	22%

Abbreviation: HTA, health technology assessment.

Nineteen respondents (70%) answered the question about which HTA practice serves as best practice. Of these respondents, 21% mentioned CADTH, followed by NICE (16%).

### Step 1 – Installation of an Appraisal Committee/Stakeholder Panel

With regard to the existence of an appraisal committee/stakeholder panel and related guidance it became clear that 62% of the respondents mentioned to have such a committee installed in their country. Two respondents questioned the need for one central committee, and mentioned that it could be beneficial to have several specialised committees (eg, for drugs and medical devices) with overlapping functions and responsibilities. A publicly available guideline or document that describes the roles and responsibilities of the committee/panel (remit and scope), and the procedures followed was present according to slightly more than half of the respondents (54% and 58%, respectively). A document that describes the composition, terms, and selection of members, as well as the roles and responsibilities of stakeholders involved in the process was felt to be less often present (46%). The respondents expressed the need for guidance with respect to various elements of an appraisal committee/stakeholder panel, ranging from 38%–46% for the different elements (Table 2). Two respondents also explicitly mentioned that training of stakeholders about how to contribute to an appraisal committee/panel could be beneficial.

**Table 2:** Views Regarding the Presence of Elements Related to an Appraisal Committee/Stakeholder Panel, and the Need for Guidance (n = 26)

**Elements**		**Present**	**Present to Some Extent**	**Not Present**	**Guidance Needed**
	Existence of a committee for appraisal/HTA decision-making or a stakeholder panel	62%	35%	3%	42%
Guidelines/document – that is publicly available – describing	The composition, terms, and selection of members	46%	35%	19%	38%
	The roles and responsibilities of the committee/panel (remit and scope)	58%	27%	15%	38%
	The roles and responsibilities of stakeholders involved in the process	46%	31%	23%	46%
	The (formal) approach(es) followed by the committee/panel	54%	31%	15%	46%

Abbreviation: HTA, health technology assessment.

We also asked the respondents if they were aware of any HTA practice that could serve as best practice. Of the 19 respondents that answered this question (73%), both CADTH and NICE were mentioned each by 21%.

### Step 2 – Selecting Technologies and Criteria

The elements in relation to selecting technologies and criteria (ie, existence of an early warning system/horizon scanning, and existence of a scoping procedure) were most often not present or present to some extent, according to the majority of the respondents. Also, more than half of the respondents felt that guidance was needed for almost all specific elements; with regard to the methods used for horizon scanning 46% of the respondents had the opinion that guidance was needed (Table 3).

**Table 3:** Views Regarding the Presence of Elements Related to Selecting Technologies and Criteria, and the Need for Guidance (n = 26)

**Element**		**Present**	**Present to Some Extent**	**Not Present**	**Guidance Needed**
	Existence of an early warning system/horizon scanning system	27%	35%	38%	73%
Guidelines/document – that is publicly available – describing	The process of identification and selection of health technologies (ie, procedures, criteria)	35%	50%	15%	58%
	The roles and responsibilities of stakeholders involved in the process	23%	31%	46%	58%
	The methods used	35%	38%	27%	46%
	Existence of a scoping procedure for HTA	35%	35%	30%	54%
Guidelines/document – that is publicly available – describing	The process of scoping (ie, procedures, criteria)	31%	35%	34%	54%
	The roles and responsibilities of stakeholders involved	8%	58%	34%	65%
	The methods used	27%	38%	35%	62%

Abbreviation: HTA, health technology assessment.

Fourteen respondents (54%) answered the question regarding best practices for horizon scanning. Of these respondents, 14% mentioned the UK National Institute for Health Research Innovation Observatory, CADTH was also mentioned by 14%, as well as the Spanish cross-regional collaboration between HTA agencies in the field of non-pharmaceuticals,^16^ and EuroScan, a non-for-profit network and scientific association of public HTA agencies, scientific organizations and individuals for sharing and collecting information and development of methods for the early identification, appropriate use and awareness of health technologies.^17^ CADTH was specifically mentioned as a best practice in relation to scoping by several respondents.

### Step 3 - Assessment

The findings show that 2 out of the 3 elements related to conducting and reporting assessments are present in most of the HTA practices surveyed. The element that was mainly present to some extent concerns stakeholder consultation to review the plausibility of the evidence reports. However, the respondents overall felt that there was less need for guidance regarding the elements linked to the assessment phase, ranging from 12%–32% for the different elements (Table 4). By those who answered the question on best practices (63%), European Network for Health Technology Assessment (EUnetHTA) (HTA Core Model^18^ and methodological guidelines)was mentioned as a best practice example by 35%, followed by the Cochrane Handbook for Systematic Reviews of Interventions (12%).^19^

**Table 4:** Views Regarding the Presence of Elements Related to Conducting and Reporting Assessments, and the Need for Guidance (n = 25)

**Element**	**Present**	**Present to Some Extent**	**Not Present**	**Guidance Needed**
Publicly available guidelines/ documents on how to undertake the HTA in terms of data collection and analysis	72%	24%	4%	12%
Existence of a tool/template for reporting and summarising the (quality of the) evidence per relevant aspect as part of HTA (assessment)	76%	20%	4%	24%
Existence of approach for stakeholder consultation to review the plausibility of the evidence reports	32%	56%	12%	32%

Abbreviation: HTA, health technology assessment.

### Step 4 – Appraisal

With regard to the appraisal phase it became clear that all the surveyed elements (existence of a formal framework/approach and a publicly available document/guideline for conducting the appraisal) are present in less than half of the responding HTA agencies. More than half of the respondents mentioned that there was a need for guidance; only with regard to the process less than half (44%) of the respondents felt that guidance was needed (Table 5). Three respondents explicitly mentioned the need to receive guidance on how to involve stakeholders in the appraisal process. Sixty percent of the respondents answered the question on best practices. Of these, 20% mentioned NICE as best practice for undertaking appraisal, followed by CADTH (13%).

**Table 5:** Views Regarding the Presence of Elements Related to the Appraisal Phase, and the Need for Guidance (n = 25)

**Element**		**Present**	**Present to Some Extent**	**Not Present**	**Guidance Needed**
	Existence of formal framework/approach for appraisal/HTA decision-making	40%	48%	12%	52%
Publicly available guidelines/documents describing	The process of appraisal (ie, procedures, deliberation)	48%	28%	24%	44%
	The roles and responsibilities of stakeholders involved in the process	24%	52%	24%	56%
	The methods used	28%	36%	36%	64%

Abbreviation: HTA, health technology assessment.

### Step 5 – Communication and Appeal

Almost all respondents (92%) indicated that decisions and the underlying reasons are made public or made public to some extent, and 60% indicated that there is no need for guidance in this respect. However, guidelines or documents describing the mechanism(s) for appeal, how to propose revisions, and to receive a reasoned response, as well as addressing monitoring and evaluation of the process were less present. Not surprisingly, more than half of the respondents felt that there is a need for guidance (52% and 56%, respectively) (Table 6). With regard to best practices, NICE was mentioned by 14% of the respondents (56%) to this sub-question.

**Table 6:** Views Regarding the Presence of Elements Related to Communication and Appeal, and the Need for Guidance (n = 25)

**Element**		**Present**	**Present to Some Extent**	**Not Present**	**Guidance Needed**
	The decisions and the underlying reasons are made public	52%	40%	8%	40%
Guidelines/documents – that is publicly available – describing	The mechanism(s) for appeal, how to propose revisions, and to receive a reasoned response	24%	40%	36%	52%
	The process of monitoring and evaluation of the HTA process and the recommendations/guidance or decisions made	20%	36%	44%	56%

Abbreviation: HTA, health technology assessment.

## Discussion

This semi-structured survey intended to collect views and experiences from HTA agencies around the globe in order to identify the level of use of EDPs by HTA agencies, and to further develop the EDP guide to support HTA agencies. The level of use of EDPs was measured by asking respondents about the extent to which elements of the EDP framework were present and whether there was a need for further guidance. We found that contextual factors to support HTA development and the critical elements regarding conducting and reporting on HTA are in place. This is reflecting current HTA practice of the respondents, as most INAHTA members are already well-established HTA agencies. However, respondents indicated that guidance was needed for specific elements related to selecting technologies and criteria, appraisal, and communication and appeal. Guidance was especially felt in terms of the practical organization of meaningful stakeholder participation and the methods to include deliberation during the appraisal step. As a result, we have updated the practical guide^12^ using these insights. Specifically, we have added guidance on stakeholder participation^20^ as well as guidance on how to use MCDA for HTA agencies, based on a recent consensus statement among more than 20 experts in the field.^21^

### Study Limitations

It is important to highlight the limitations of this study. First, the response rate (54%) might suggest that some selection bias might be present. However, the survey respondents reflect the INAHTA membership (in 2018) in terms of geographical representation. As such we feel confident that some conclusions can be drawn from this study, even though we acknowledge that reports by single persons from an INAHTA member agency are not representing the overall HTA practice in a particular jurisdiction. In addition, bias might be that the responses came from HTA agencies that are already doing quite well, while the survey might not have captured responses from less well-established HTA agencies. This might have led to an underestimation of the percentage of HTA agencies that actually are in need of guidance. We are aiming to broadening the survey towards HTA organizations from low- and middle-income countries. Second, reporting on any set of elements implies that they are equally important, but this is not true. Depending on the contextual factors, certain steps and/or elements can be more important than others. Therefore the findings should be mainly viewed as indicative for the level of EDP use by HTA agencies.

### Alignment With Other Study Findings

The findings suggest that contextual factors to support HTA development and the critical elements regarding conducting and reporting on HTA are currently overall present, mainly in countries with well-established HTA practices. Furthermore, respondents indicated that specific guidance was needed for elements related to selecting technologies and criteria, including scoping, appraisal, and communication and appeal. These results are in line with the findings of the ISPOR HTA Council that recently presented a report on good practices in HTA.^1^ From this report it becomes clear that many good practices have been developed in the areas of assessment (Step 3), and several with regard to priority setting, scoping (Step 2), only a few with regard to structure, governance, organizational aspects (Step 1), deliberative processes (Step 4), and measuring the impact (Step 5). The findings are also in line with previous work on priority setting^22,23^ and reported needs of HTA agencies in specific regions. For example, in Latin America, the need for transparency in the production of HTA, involvement of relevant stakeholders in the process, mechanisms to appeal decisions, clear priority-setting processes, and a clear link between HTA and decision-making have recently been emphasized.^24,25^ In addition, HTA agencies in Asia recently expressed their need to improve transparency and accountability throughout the process. For example, it was recommended by the HTAi Asia Policy Forum members that a standardized, transparent methodology for priority-setting regarding coverage decision-making needs to be developed.^26^ Furthermore, some respondents explicitly mentioned particular best practices per step, such as the CADTH for scoping, and NICE for having clear procedures in place for appeal. Also, Brazil was mentioned as best practice for the Latin American region. These findings are in line with previous studies in this area.^3,27^ As such, we feel empowered to further optimize the guide on EDPs to support HTA agencies.

## Conclusion

This is the first survey among HTA agencies regarding the use of EDPs and provides useful information for further developing a practical guide for HTA agencies around the globe. Based on the results from the survey, we conclude that – as expected – several HTA agencies have already certain (elements of) EDPs in place and can serve as inspiration for others. The results could also serve as a baseline measurement for HTA agencies for future monitoring and evaluation of the level of EDP use and to study the effectiveness of EDPs in practice. This could support them in improving their processes and enhancing legitimate decision-making.

## Acknowledgements

We thank Agnes Toll for supporting us in developing the online survey. We thank the INAHTA secretariat for their support in distributing the invitation to participate in the survey, and we are grateful to the survey respondents for providing their views on the needs of HTA agencies regarding optimizing their processes and listing best practices.

## Ethical issues

Not Applicable. We contacted the secretariat of the INAHTA for supporting us in contacting their members. The secretariat provided consent and sent an invitation to participate in the survey to their members through e-mail. Reminders were sent by WO, targeting the contact persons as listed on the INAHTA website, which is publicly available. In the introduction to the survey, we made clear that all answers were treated confidential: no attribution would be made to specific persons. Two survey respondents explicitly wished not to disclose their affiliation, we therefore provide their results anonymously.

## Competing interests

None. The work described in the manuscript is conducted as part of a research program that rethinks the theory and tools to value interventions for priority setting in healthcare (REVISE2020), and is supported by a grant from the Netherlands Organisation for Scientific Research (NWO).

## Authors’ contributions

All authors were involved in the conception of the study. WO and MJ were involved in the design of the study and data collection. WO analysed the data and drafted the manuscript. All the authors read and approved the final version of the manuscript.

## Supplementary files


Supplementary file 1 contains the questionnaire.

## Key messages

**Implications for policy makers**

- The evidence-informed deliberative processes (EDPs) framework can support the decision-making process of health technology assessment (HTA)-agencies but is also relevant for countries that have not (yet) established such an agency. It takes the current decision-making context as the starting point, and offers specific advice depending on the level of HTA development.
- Using EDPs can contribute to the legitimacy of recommendations and/or decisions, eg, by improving the quality, consistency and transparency of the HTA process.
- The results provide an overview of the level of use of EDPs by HTA agencies around the globe. It includes best practices for the different parts of the EDP framework, and as such the results are practice-oriented and meant to be inspirational to improve HTA practices.

**Implications for public**

Health technology assessment (HTA) is intended to inform decision-making, including decisions regarding which health technologies (eg, drugs, medical devices, surgical procedures, vaccination programs) should be reimbursed or not (anymore). There is a need for structured, explicit and transparent approaches with regard to how such decisions are made to facilitate legitimate decision-making. Current HTA methodologies and decision-making informed by HTA only partly respond to these requirements. Using evidence-informed deliberative processes (EDPs) can support this; by enhancing stakeholder deliberation throughout the HTA process it contributes to the legitimacy of recommendations and/or decisions. This manuscript provides insight in *(a)* how HTA agencies ideally should organise their processes in line with EDPs, which could include the involvement of citizens and their views; and *(b)* to what extent this is currently being implemented by members of the International Network of Agencies for Health Technology Assessment (INAHTA).
