# Supplementary material for: Use of Evidence-Informed Deliberative Processes by Health Technology Assessment Agencies Around the Globe
Source: Int J Health Policy Manag. 2019 Sep 15;9(1):27–33. doi: 10.15171/ijhpm.2019.72 (PMC6943303; doi:10.15171/ijhpm.2019.72)
Supplement: Supplementary file 1 — contains the questionnaire. [file ijhpm-9-27-s001.pdf]

Supplementary file 1. Survey

Welcome to the survey on the potential use of 'evidence-informed deliberative processes' (EDPs) by HTA organizations. EDPs provide guidance to HTA organizations to improve their processes towards more legitimate decision-making.

Completing this survey will take approximately 20 minutes as most of the questions only require you to tick a box.

Please note that all your answers will be treated confidential: no attribution will be made to specific persons.

General information

What is your name and title?
What is your email address?
What is the name of your organization/department?
What country or region are you representing?

Introduction to EDPs

EDPs provide guidance to HTA organizations to improve their processes towards more legitimate decision-making. The framework is not entirely new, and integrates two increasingly popular and complementary approaches to HTA informed decision-making. EDPs are based on **rational decision-making** through evidence-informed evaluation of identified values (as reflected in multi-criteria decision analyses) as well as **fair decision-making** (as reflected in the accountability for reasonableness approach – A4R). In other words, EDPs focus on **stakeholder deliberation throughout the HTA process** to learn about the importance of relevant values.

We distinguish several steps in the use of EDPs (Figure 1), and define its elements on the basis of literature, and existing checklists such as the INAHTA checklist for HTA reports. The way in which these steps can be applied depends on the context.
We are aware that some HTA organizations already have (some of) the processes in place, and they may serve as inspiration for others. There may be other organizations that can substantially improve their processes. In order to increase our understanding of the potential use of EDPs in HTA organizations, to identify topics that are in need of more guidance, and to learn about best practices in relation to each step, we would like to collect your views and experiences.

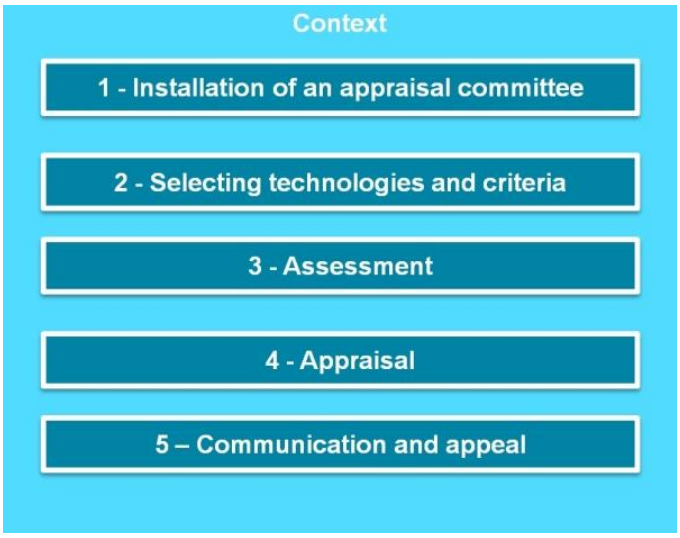

Figure 1. The steps of EDPs.

Context

It is important to understand the context of a HTA practice, as this influences the implementation of EDPs.

QUESTION:

Please could you indicate a) if any of the following factors are ***Present, Present to some extent or Not present*** in your country/region and b) whether particular factors are in need of guidance in your country/region ***(Yes/No)***:

Linkage between HTA and policy/practice

- A (formal) mechanism or process to link HTA to policy making (eg, legislation)

- Allocation of public ***funding*** to HTA on an annual basis
- A policy statement on the ***willingness to use*** HTA in policy and/or practice

Institutional environment

- An independent ***organizational structure*** and/or institutional set-up for HTA (HTA organization or HTA focal point)
- HTA ***process guidelines*** (is a systematic process in place for eg, assessment and appraisal)
- HTA ***method guidelines*** (is methodological guidance available for eg, conducting economic analysis or clinical assessment)

Networking and capacity

- An (inter)national ***networking strategy*** for collaboration between HTA organization(s) and relevant stakeholders
- Sufficient ***capacity*** to carry out HTA, including medical disciplines, public health specialists, epidemiologists, statisticians, psychologists, biomedical engineers and/or economists
- Ability to ***review international literature*** (ie, access to databases), including expertise in searching the internet
- (Domestic) ***HTA training opportunities*** (short courses, workshops, Master programmes and PhD training)

QUESTION:
Please could you indicate below a) if any important contextual factor has not been listed and b) whether these areas are in need of guidance in your country/region:

QUESTION:
With regard to all contextual factors listed in the questions above, are you aware of any HTA practice that can serve as best practice? If so, please can you provide more information?

Step 1. Installation of an appraisal committee / stakeholder panel

Most HTA practices have an appraisal committee or stakeholder panel in place. Such committee or stakeholder panel can be involved throughout the entire HTA process, including scoping (step 2), assessment (step 3), and appraisal (step 4). In some HTA practices, the committee or panel develops recommendations or guidance, in other contexts it is responsible for taking decisions.

It is increasingly acknowledged that the members of such a committee or panel should represent societal perspectives. For this purpose, it is advised to set-up an (independent) committee or stakeholder panel including relevant stakeholders (eg, clinicians, patients, citizens, payers, providers, decision-makers). For reasons of legitimacy, it is important that at least the composition, selection of members, the terms, its independence (eg, COI statement), remit and scope are explicit and transparent.

QUESTION:
Please could you indicate a) if any of the following elements are ***Present, Present to some extent or Not present*** in your country/region and b) whether particular elements are in need of guidance in your country/region ***(Yes/No)***:

- Existence of a committee for appraisal/HTA decision making or a stakeholder panel

- Guidelines / document – that is publicly available - describing:
    - The composition, terms, and selection of members;
    - The roles and responsibilities of the committee / panel (remit and scope);
    - The roles and responsibilities of stakeholders involved in the process;
    - The (formal) approach(es) followed by the committee/panel.

- Please could you indicate below a) if any important element has not been listed and b) whether these areas are in need of guidance in your country/region:

- With regard to all elements related to an appraisal committee or stakeholder panel listed in the questions above, are you aware of any HTA practice that can serve as best practice? If so, please can you provide more information?

Step 2. Selection of technologies and criteria

The selection of health technologies in need for HTA is an important step in the HTA process. This requires standardised systems for the identification of health technologies through eg, horizon scanning or an early warning system, and the prioritization of these health technologies on the basis of agreed criteria or guidelines.

Subsequently, for selected health technologies, HTA organizations should define the scope of the assessment. This includes a clearly defined policy question(s) of direct significance to decision-makers and the identification of criteria for which evidence needs to be collected. Scoping is ideally done in consultation with relevant stakeholders, eg, patients, informal carers, and health professionals. Often HTA organizations are responsible for the scoping procedure, but this can also be done by policy makers, Ministry of Health, external committees, in consultation with relevant stakeholders and/or experts.

QUESTION:

Please could you indicate a) if any of the following elements are ***Present, Present to some extent or Not present*** in your country/region and b) whether particular elements are in need of guidance in your country/region ***(Yes/No)***:

- Existence of an **early warning system / horizon scanning system**
- Guidelines / document – that is publicly available - describing:
    - The process of identification and selection of health technologies (ie, procedures, criteria);
    - The roles and responsibilities of stakeholders involved in the process;
    - The methods used.

- Existence of a **scoping** procedure for HTA

- Guidelines / document – that is publicly available - describing:
    - The process of scoping (ie, procedures, criteria);
    - The roles and responsibilities of stakeholders involved;
    - The methods used.
- Please could you indicate below a) if any important element has not been listed and b) whether these areas are in need of guidance in your country/region:

- With regard to all elements related to the identification and selection of health technologies listed in the questions above, are you aware of any HTA practice that can serve as best practice? If so, please can you provide more information?

Step 3. Assessment

*The assessment process consists of evidence collection, analysis of the quality of the evidence, synthesising the evidence, and reporting the findings and implications. HTA organizations should ideally develop an elaborated HTA protocol to plan this process***,** *based on the policy question(s) defined during scoping (step 2). Several guidelines exist for data collection and analysis (eg, methodological guidance such as the EUnetHTA core model, INTEGRATE-HTA, as well as country/agency-specific guidelines on how to conduct the assessment – eg, NICE, CADTH, PBAC; and GRADE for assessing the quality of the evidence). Ideally, HTA organizations should present the collected evidence in evidence reports and standardised evidence summaries for each relevant assessment aspect (defined during scoping). The reports should ideally be subjected to an independent review and discussed by relevant stakeholders.*

QUESTION:

Please could you indicate a) if any of the following elements are ***Present, Present to some extent or Not present*** in your country/region and b) whether particular elements are in need of guidance in your country/region ***(Yes/No)***:

- Publicly available guidelines / documents on how to undertake the HTA in terms of data collection and analysis

- Existence of a tool/template for reporting and summarising the (quality of the) evidence per relevant aspect as part of HTA (assessment)

- Existence of approach for stakeholder consultation to review the plausibility of the evidence reports

- Please could you indicate below a) if any important element has not been listed and b) whether these areas are in need of guidance in your country/region:

- With regard to all elements related to the assessment of health technologies listed in the questions above, are you aware of any HTA practice that can serve as best practice? If so, please can you provide more information?

Step 4. Appraisal

In the appraisal step, the HTA organization interprets the results of the assessment and formulates a recommendation or guidance to inform decision makers. In several HTA organizations, this task is carried out by a specific committee (step 2). Ideally, such a committee follows an explicit approach which guarantees the quality, consistency and transparency of the recommendations, guidance or decisions. The approach should preferably include a deliberative component for the interpretation of the assessment results, and involvement of relevant stakeholders to allow the inclusion of their perspectives in the development of recommendations, guidance or decisions. Several methods are available to support HTA organizations in this process, such as multi-criteria decision analysis or qualitative decision tools.

QUESTION:

Please could you indicate a) if any of the following elements are ***Present, Present to some extent or Not present*** in your country/region and b) whether particular elements are in need of guidance in your country/region ***(Yes/No)***:

- Existence of formal framework/approach for appraisal/HTA decision making

- Publicly available guidelines / documents describing:
    - The process of appraisal (ie, procedures, deliberation);
    - The roles and responsibilities of stakeholders involved in the process;
    - The methods used.

- Please could you indicate below a) if any important element has not been listed and b) whether these areas are in need of guidance in your country/region:

- With regard to all elements related to the appraisal of health technologies listed in the questions above, are you aware of any HTA practice that can serve as best practice? If so, please can you provide more information?

Step 5. Communication and appeal

Communication and appeal are important features that enhance the legitimacy of decision making. These concepts are part of the A4R framework, which states that the decisions should be made public as well as the argumentation and evidence on which decisions are based. In addition, there must be a mechanism in place that gives stakeholders the possibility to appeal against decisions, propose revisions, and receive a reasoned response. This should give decision makers the chance to reconsider their decisions using extra (new) arguments or (new) evidence.

QUESTION:

Please could you indicate a) if any of the following elements are ***Present, Present to some extent or Not present*** in your country/region and b) whether particular elements are in need of guidance in your country/region ***(Yes/No)***:

- The decisions and the underlying reasons are made public

- Guidelines / document – that is publicly available – describing the mechanism(s) for appeal, how to propose revisions, and to receive a reasoned response

- Guidelines / document – that is publicly available – describing the process of monitoring and evaluation of the HTA process and the recommendations/guidance or decisions made
- Please could you indicate below a) if any important element has not been listed and b) whether these areas are in need of guidance in your country/region:

- With regard to all elements related to communication and appeal listed in the questions above, are you aware of any HTA practice that can serve as best practice? If so, please can you provide more information?

Further comments

QUESTION:
If you wish to make any further comments about your experiences with EDPs in your country and/or this survey, please use the space provided below

May we contact you if we have additional questions? ***(Yes/No)***
